# Supplementary material for: Integrative Multi-omics Analysis of the Human Skeletal Muscle Response to Endurance or Resistance Exercise: Findings from the Molecular Transducers of Physical Activity Consortium (MoTrPAC)
Source: bioRxiv. 2026 Mar 6:2026.03.04.705181. Preprint. [Version 1] doi: 10.64898/2026.03.04.705181 (PMC13001352; doi:10.64898/2026.03.04.705181)
Supplement: Supplement 1 [file media-1.pdf]

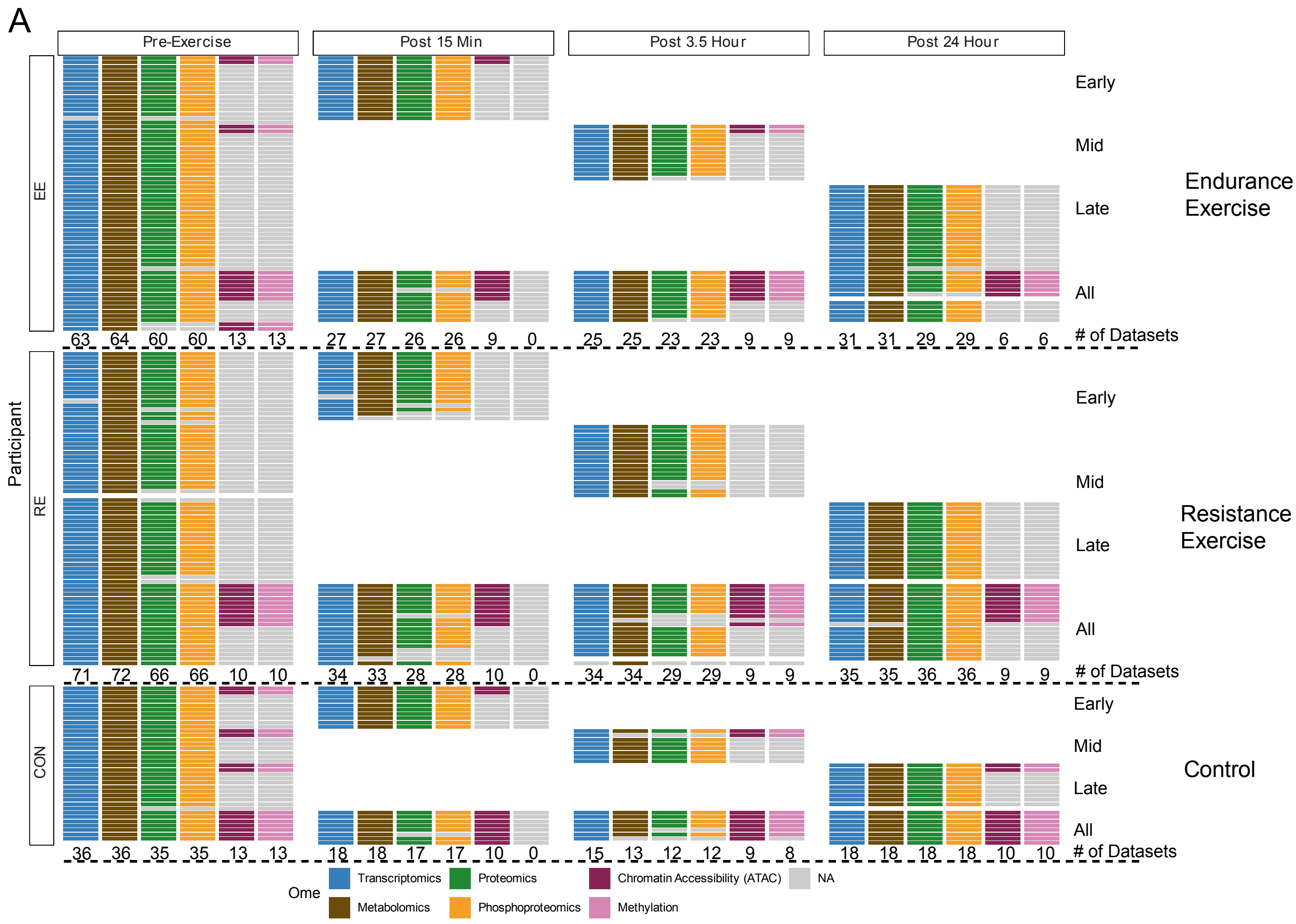

Figure S1. Samples analyzed by all omes, related to Figure 1

A. Omics Dataset Availability. Each row represents a participant. If data is available for a particular participant for a given tissue-ome-time point combination, this is indicated by a colored cell. A gray cell indicates that no omic data was available or that no sample was collected for that participant at that time point. Numbers below indicate total in each group and ome.

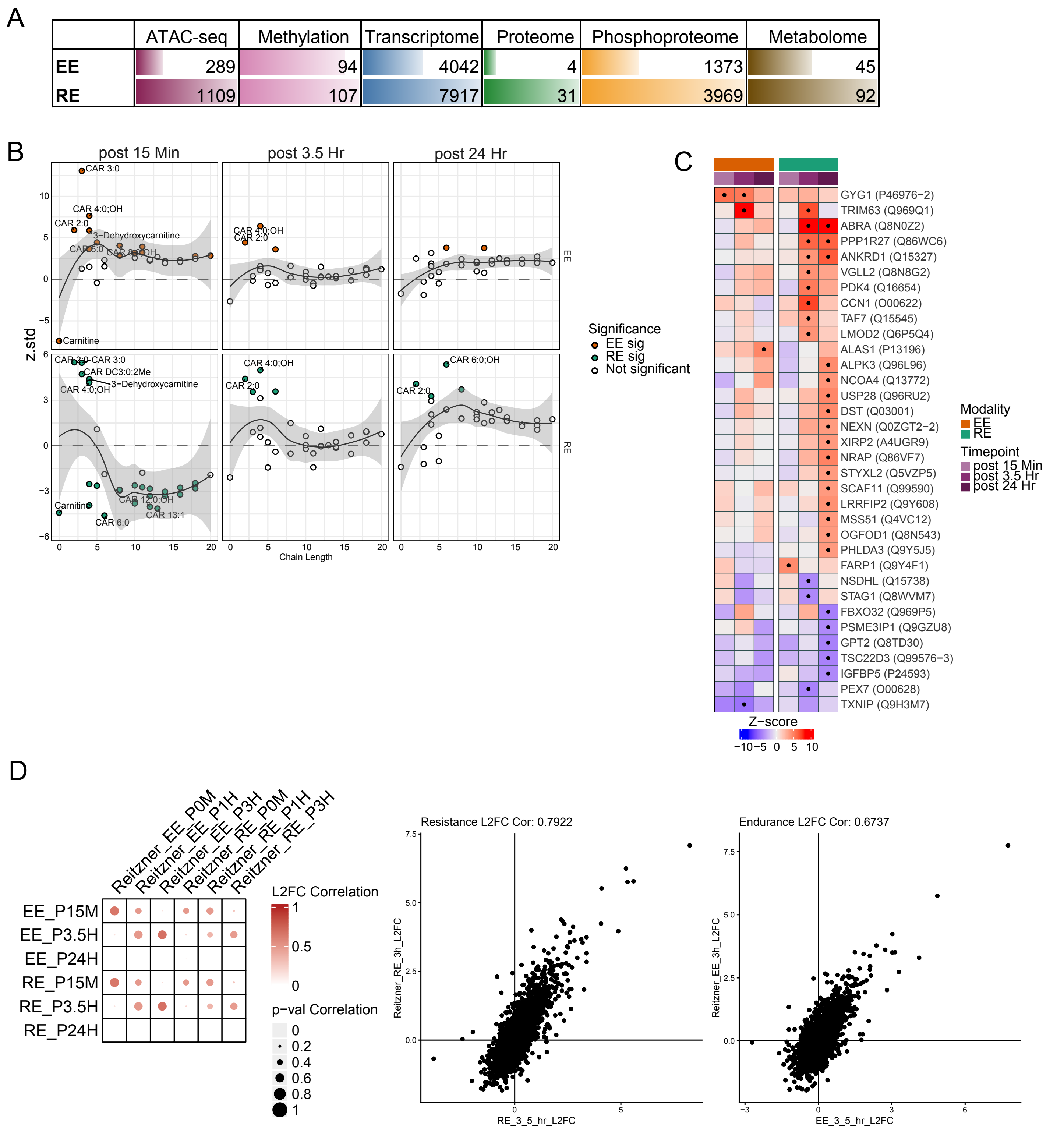

Figure S2. Differential regulation of multi-omics dataset, related to Figure 2

A. Total number of differentially abundant (DA) features across the post-exercise timepoints for each ome and exercise modality. Numbers represent the total DA features across the time points; bars are colored using ome-specific colors.
B. Upset plot illustrating overlap of differential features across the omes. DA features for ATAC-seq, methylCap, RNA-seq, proteome and phosphoproteome were mapped to the gene level for this analysis. Therefore, metabolome is not included in this analysis.
C. Z-scores of acyl carnitines (CAR) plotted by chain length, shown separately for EE and RE across post-exercise timepoints.

D. Heatmap displaying temporal dynamics of all DA proteins across post-exercise timepoint (n = 4 in EE, n = 31 in RE). Asterisks indicate significance at adj. P-value < 0.05.
E. Comparison of study transcriptomic results with previous[21]. Left: the bubble heatmap, each grid point describes correlations of L2FC and adjusted p-value between post exercise time points in each study. Size of the bubble reflects Pearson correlation of adjusted p-value of exercise versus control comparisons and the color of the bubble reflects Pearson correlation of L2FC of exercise vs control comparisons. Right: scatter plots are also included plotting L2FC of this study versus Reitzner et al study comparing EE vs Control and RE vs Control at the 3.5 hour time point, with Pearson correlations of L2FC values listed above each plot.

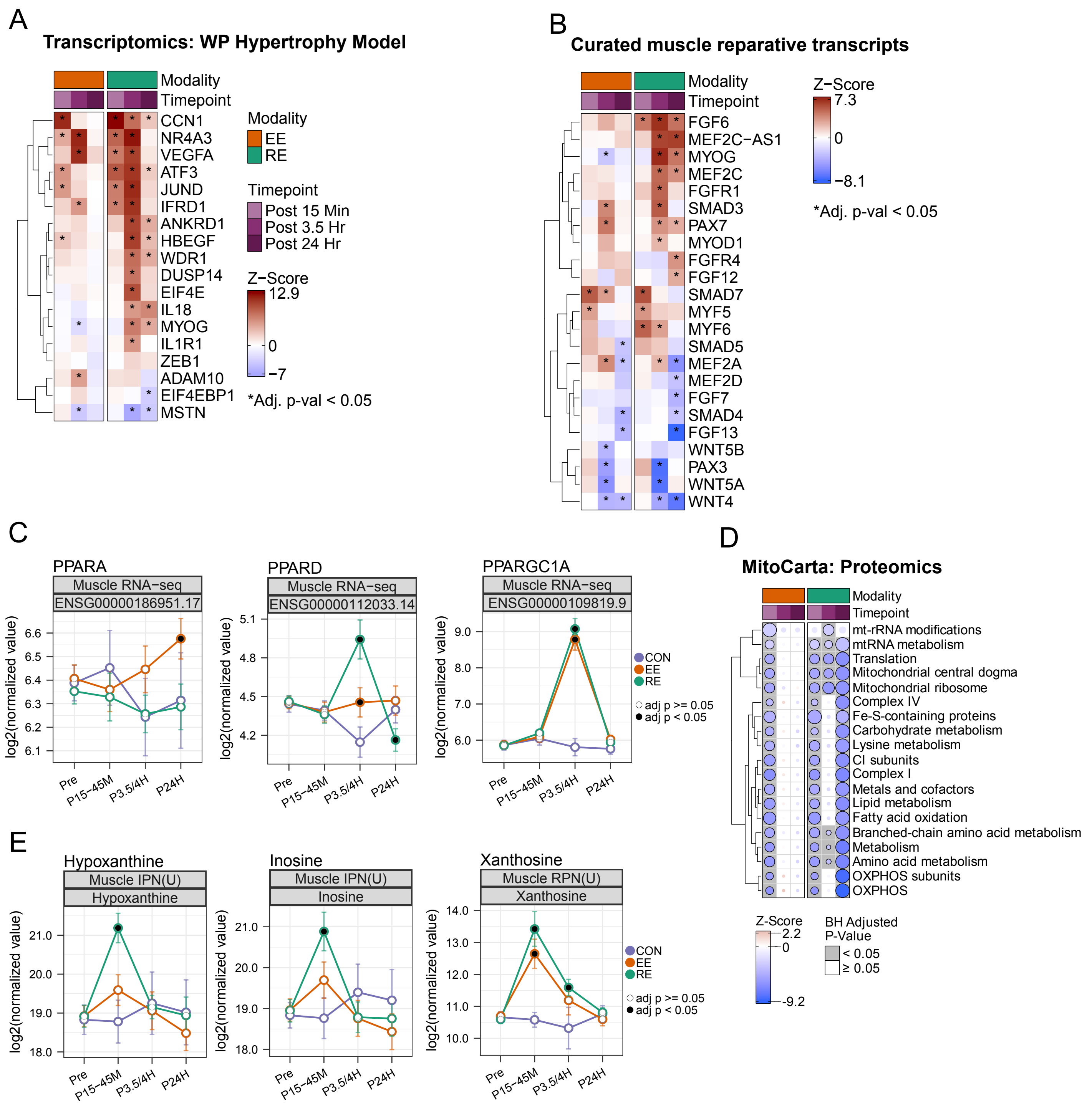

Figure S3. Pathway enrichment analysis, related to Figure 3

A. Feature level heatmap of enriched transcripts in the WP Hypertrophy Model pathway, presented in Figure 3A. Asterisks indicate individual feature significance with adj. P-value < 0.05.

B. Temporal trajectories of PPAR (Peroxisome Proliferator-Activated Receptor) transcripts: PPARA, PPARD, and PPARGC1A. EE = Endurance exercise (orange line), RE = resistance exercise (green line), CON = control group (purple), Pre = pre-exercise, D15M =15 minutes post exercise, P3.5H = 3.5 hours post exercise, P24H = 24 hours post exercise. Significance (adj. P value < 0.05) indicated by black dot per timepoint per group.

C. Heatmap of CAMERA-PR enrichment of proteomics dataset using MitoCarta 3.0 database displaying significantly enriched terms (adj. P-value < 0.05).

D. Temporal trajectory of select metabolites in the skeletal muscle. EE = Endurance exercise (orange line), RE = resistance exercise (green line), CON = control group (purple), Pre = pre-exercise, D15M =15 minutes post exercise, P3.5H = 3.5 hours post exercise, P24H = 24 hours post exercise. Significance (adj. P value < 0.05) indicated by black dot per timepoint per group.
E. Feature level heatmap of the RefMet Acylcarnitine pathway enrichment displayed in Figure 3D. Asterisks indicate significance of a given metabolite per timepoint and contrasts with adj. P-value < 0.05.

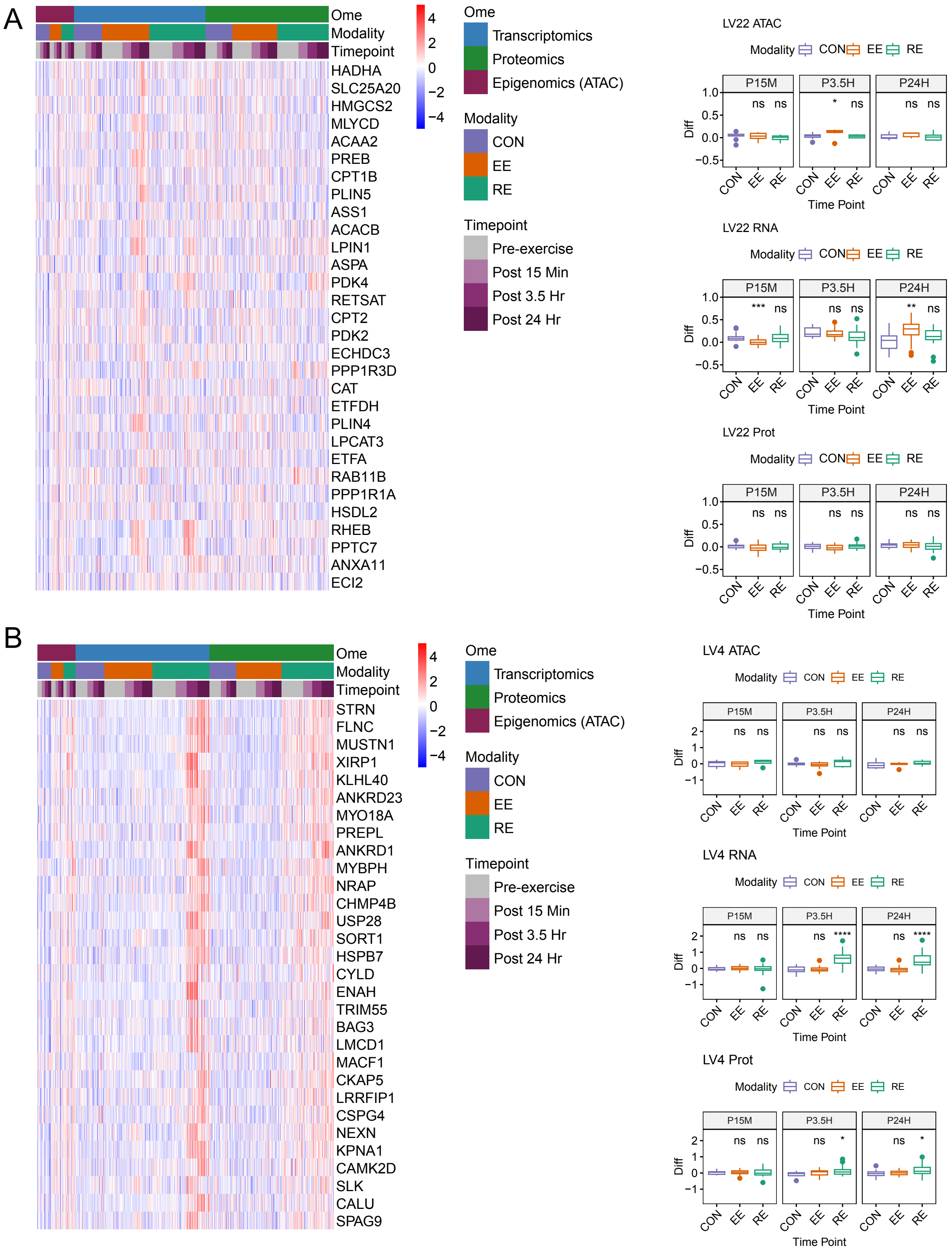

Figure S4: Cross-omic coordinated responses to exercise highlighting changes in protein abundance, related to Figure 4

A-B. Heatmaps and associated boxplots for L22 (A), LV4 (B), where the temporal pattern of omic enrichments are indicated above each panel. Heatmaps indicate within-ome z-scores of the top 30 features for each participant at every ome-time point for a given LV. Each heatmap is paired with boxplots detailing the within-subject expression level difference relative to pre-exercise of each LV in ATAC, RNA, and protein at each post-exercise time point within each modality. Significance is calculated by a t-test measuring EE or RE post- vs

pre-exercise differences relative to control subject post- vs pre-exercise differences in LV level. Significance labels represent: ns: p > 0.05, *: 5e-03 < p <= 0.05, **: 5e-04 < p <= 5e-03, ***: 5e-05 < p <= 5e-04, ****: p < 5e-05.

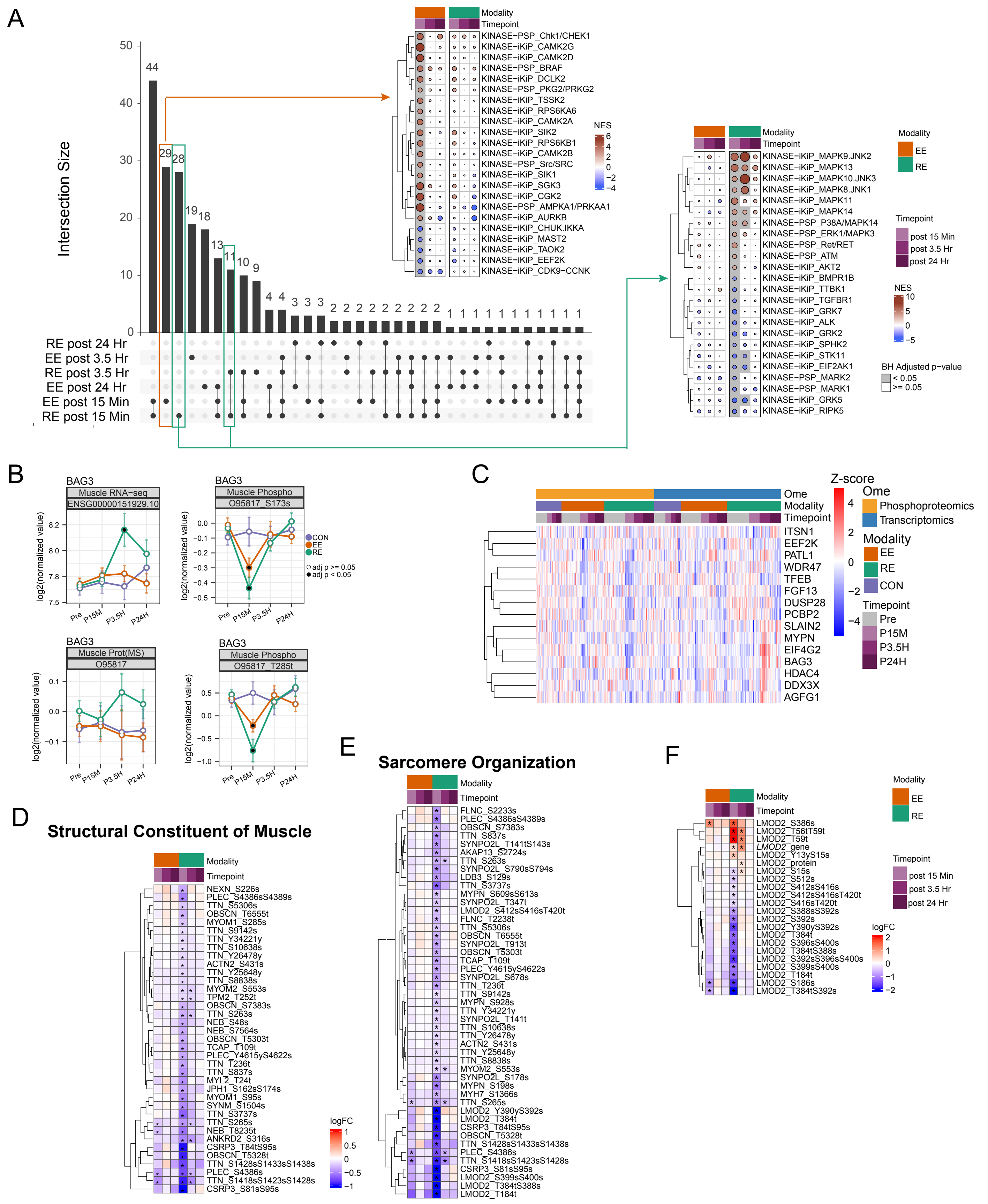

Figure S5: Phosphoproteomics response to acute exercise, related to Figure 5

A. Upset plot (left panel) illustrating overlap of significant PTM-SEA (Post Translational Modification Signature Enrichment Analysis) signatures across the exercise modalities and time points. Heatmaps

show signatures that are significant only in EE 15 minute time point (middle panel) and only in RE at 15 minute time point and shared between 15 minute and 3.5 hour time points (right panel).

B. Temporal trajectory of BAG3 (BAG family molecular chaperone regulator 3) transcript, protein and pS173 and pT285 phosphosites. EE = Endurance exercise (orange line), RE = resistance exercise (green line), CON = control group (purple), Pre = pre-exercise, D15M =15 minutes post exercise, P3.5H = 3.5 hours post exercise, P24H = 24 hours post exercise. Significance (adj. P value < 0.05) indicated by black dot per timepoint per group.
C. Heatmap of one of the LVs in the PLIER analysis with the transcriptome and phosphoproteome. Heatmap indicates within-ome z-scores of the top 15 features for each participant at every ome and time point.
D. Heatmap of GOBP Sarcomere organization pathway, one of the significantly enriched pathways in the ORA of HIPK3 pY359 nearest neighbor analysis. Asterisks indicate significance in the phosphoproteome dataset with adj. P-value < 0.05.
E. Heatmap of GOBP Structural constituent of muscle pathway, one of the significantly enriched pathways in the ORA of HIPK3 pY359 nearest neighbor analysis. Asterisks indicate significance in the phosphoproteome dataset with adj. P-value < 0.05.
F. Heatmap of differential LMOD2 (Leiomodin-2) features (transcript, protein and several phosphosites). Asterisks indicate individual feature significance with adj. P-value < 0.05.

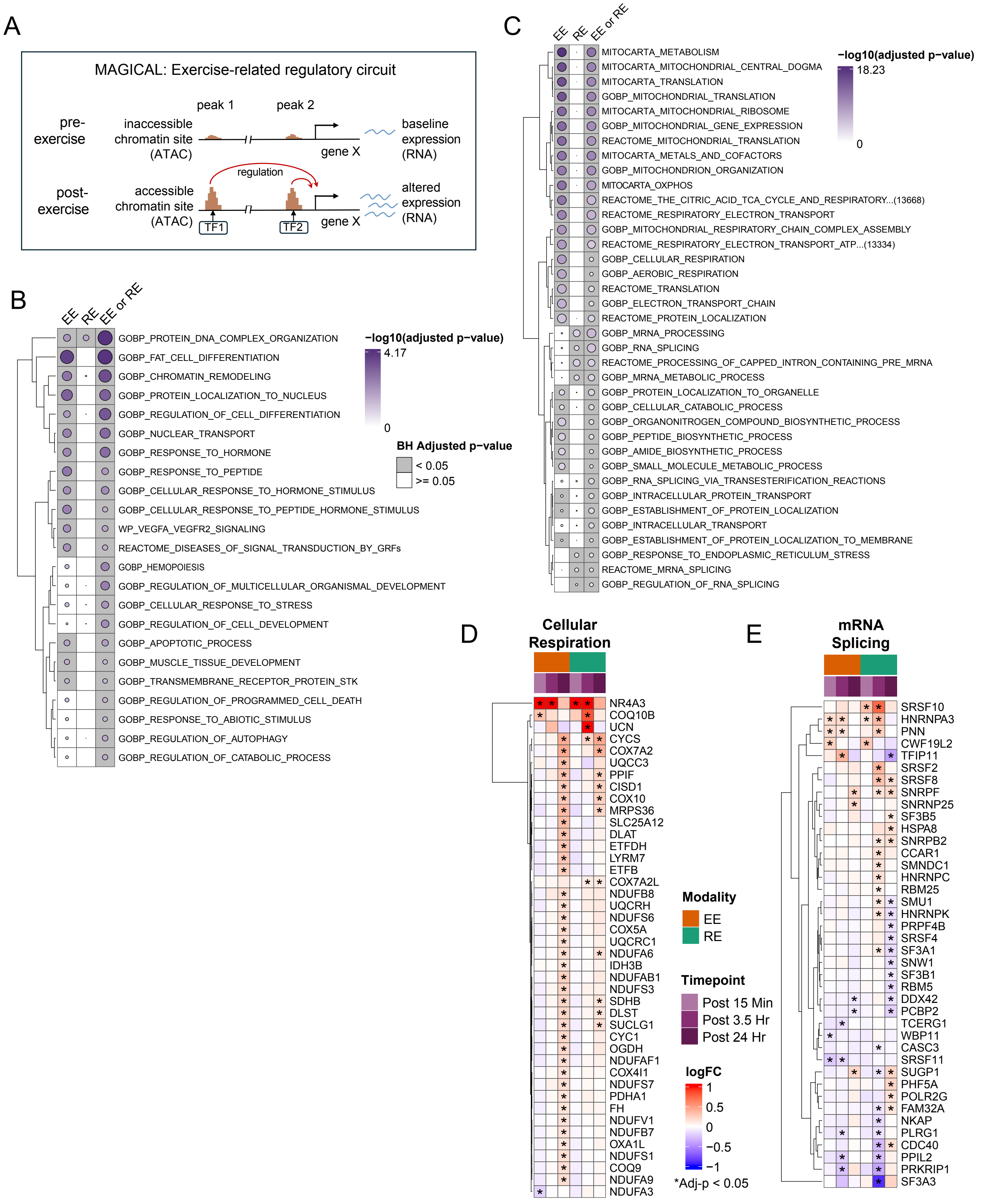

Figure S6: Integrative network analysis identifies NFIC and MEF2A as exercise response hubs, related to Figure 6

A. Example of an exercise-regulatory circuit based on ATAC-seq and transcriptome data that would be inferred by MAGICAL.

B. ORA on ChIP-supported MEF2A targets inferred using EE data, RE data, or either (EE or RE). Circle size and color represent $-\log_{10}$(adjusted p-value) and gray squares indicate adjusted p-value < 0.05.
C. ORA on ChIP-supported NFIC targets inferred using EE data, RE data, or either (EE or RE). Circle size and color represent $-\log_{10}$(adjusted p-value) and gray squares indicate adjusted p-value < 0.05.
D. Transcriptome expression of ChIP-supported NFIC targets which are in the GOBP Cellular Respiration gene set. Asterisks indicate individual feature significance with adj. P-value < 0.05.
E. Transcriptome expression of ChIP-supported NFIC targets which are in the GOBP mRNA Splicing gene set. Asterisks indicate individual feature significance with adj. P-value < 0.05.

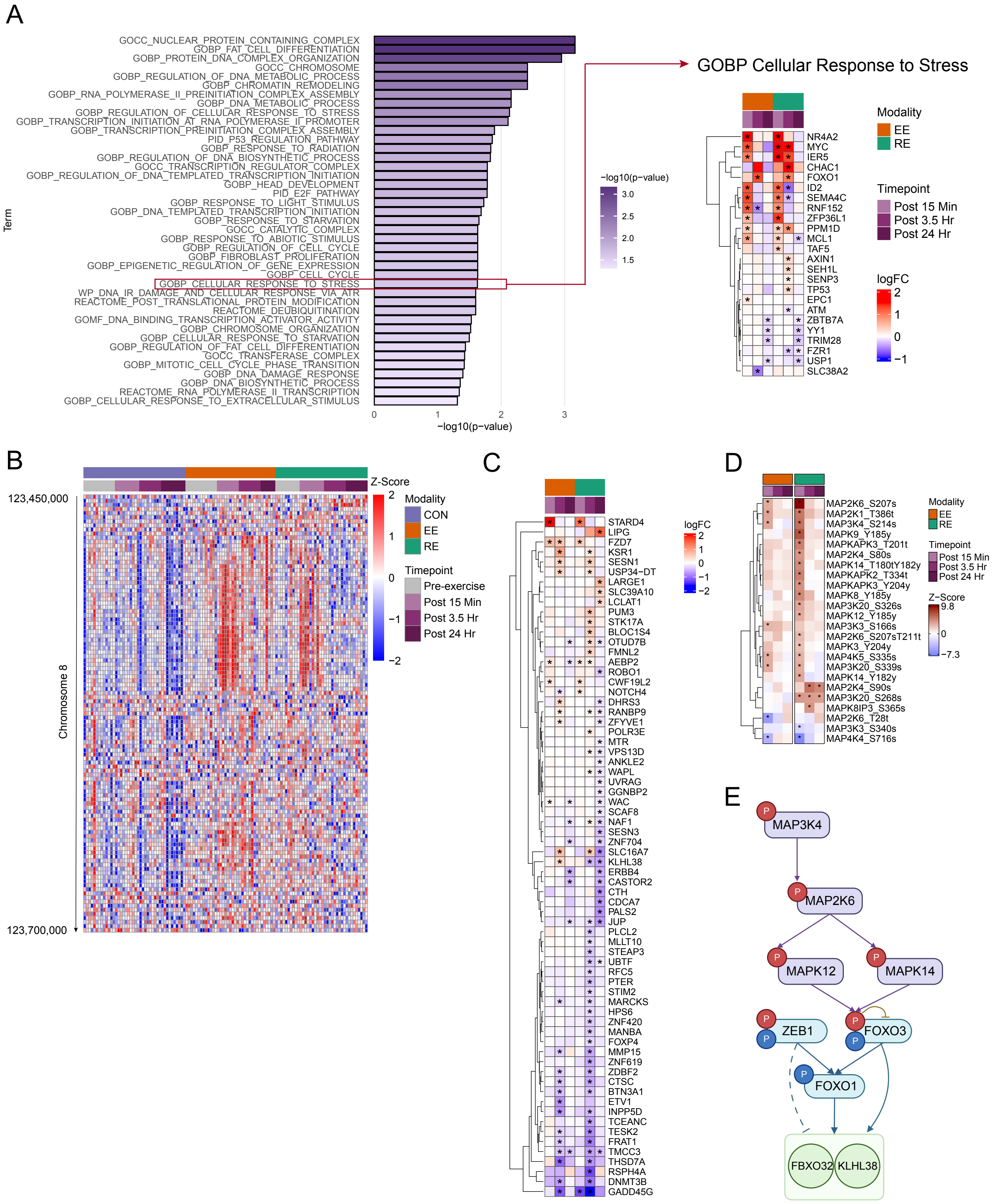

Figure S7: Coordinated regulation of muscle protein turnover, related to Figure 7

A. ORA of differentially expressed ChIPseq validated ZEB1 transcriptional targets affected by exercise (left) and exemplary feature level heatmap of GO-BP Cellular response to stress pathway displaying

modality shared and divergent ZEB1-targeted gene expression (right); asterisks indicate individual feature significance with adj. P-value < 0.05.

B. Heatmap of z-scored chromatin accessibility across all muscle samples for ATACseq peaks on chromosome 8 between base pairs 123,450,000 and 123,700,000. Samples are annotated by exercise modality and time point. Highly correlated peaks (as seen in Fig. 7D) annotated to FBXO32 show strong increases in accessibility 15 minutes post EE and RE.
C. Feature level heatmap of FOXO1 transcriptional targets affected by exercise filtered to an adj. p-value <0.01.
D. Heatmap of all MAPK phosphosites that are significantly changing (adjusted p-value < 0.05) in at least one time point following acute exercise.
E. Proposed pathway detailing the regulation of atrophy-linked genes FBXO32 and KLHL38. Phosphosite colors represent response to exercise (red: up, blue: down). MAP3K4 phosphorylates MAP2K6, which phosphorylates MAPK12 and MAPK14, both of which phosphorylate a site on FOXO3 known to inhibit and degrade FOXO3 activity. We identify FOXO3 and ZEB1 as coregulators of FOXO1, all three of which act on FBXO32 and KLHL38. The combination of RE-induced ZEB1 inhibition and the degradation of FOXO3's enhancement of the target genes leads to the decrease of FBXO32 and KLHL38 expression post RE, despite their increase post EE.
